# Transient Second-Degree Atrioventricular Block: A Rare Finding in Sickle Cell Crisis

**DOI:** 10.7759/cureus.9579

**Published:** 2020-08-05

**Authors:** Sana Riaz, Parth J Sampat, Rajat Dhungana, Luna Bhatta

**Affiliations:** 1 Internal Medicine, State University of New York Upstate Medical University, Syracuse, USA; 2 Cardiology, State University of New York Upstate Medical University, Syracuse, USA

**Keywords:** mobitz type 1 av block, sickle cell crisis, cardiac arrhythmias, sickle cell disease

## Abstract

Sickle cell disease (SCD) affects approximately 100,000 Americans, and it occurs in one out of every 365 African American births. Cardiac complications are a common feature of SCD and are an essential cause of the morbidity and mortality associated with SCD. However, there is insufficient literature on SCD and atrioventricular (AV) blocks. We present a case of a young African American male with transient second-degree Mobitz type 1 block during an acute episode of sickle cell crisis.

## Introduction

Sickle cell anemia is an often-overlooked cause of transient atrioventricular (AV) conductive disorders. There is insufficient data on the correlation between sickle cell anemia and rhythm disturbances [1]. We want to highlight the rarely reported complication with sickle cell crisis through our case of a 21-year-old male with known homozygous sickle cell disease (SCD) with Mobitz type 1 AV block. 

## Case presentation

**Figure 1:**
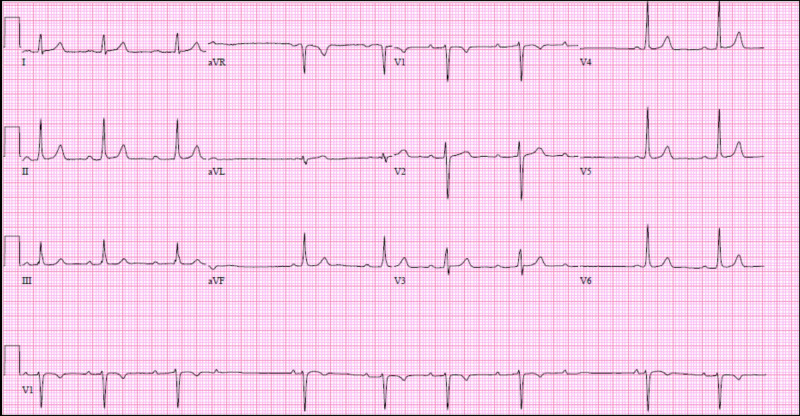
Electrocardiogram demonstrating Mobitz type 1 atrioventricular block

A 21-year-old African American male with a past medical history of SCD (severe hemoglobin SS) dependent on chronic transfusions and maximum dose of hydroxyurea and repeated episodes of sickle cell crisis presented with acute-onset substernal chest pain. He denied symptoms of dizziness, syncope, and palpitations. Vital signs were notable for blood pressure 130/90 mmHg and heart rate ranged between 55 and 70 bpm. Laboratory work-up showed mild elevations in total bilirubin (2.2 mg/dL), direct bilirubin (0.3 mg/dL), hemoglobin 9.4 mg/dL, and hematocrit 28.2%. An electrocardiogram (EKG) obtained due to his ongoing chest pain demonstrated sinus rhythm with intermittent second-degree Mobitz type 1 AV block (Figure 1). Echocardiogram showed normal left ventricular (LV) wall thickness and LV ejection fraction of 60%-65%. EKG in previous admissions showed sinus rhythm, and there was no evidence of AV block. During the admission, he was managed for sickle cell crisis with blood transfusion, hydration, and pain medications. He was monitored on telemetry, and it continued to show intermittent episodes of Mobitz type 1 AV block, but he remained asymptomatic. 

Upon stabilization, he was discharged home after eight days of hospitalization. Three months after initial hospitalization, repeat hospital admission for sickle cell crisis no longer showed AV block on EKG. 

## Discussion

SCD is a point mutation in the beta-globin chain that causes the substitution of glutamic acid with valine at position 6. This hemoglobin is referred to as hemoglobin S. Deoxygenation of hemoglobin S causes polymerization that reduces the flexibility of red blood cells and causes red blood cell rigidity that can lead to obstruction in the microvasculature, thereby causing ischemia-induced injury to several vital organs, including bone, muscle, brain, kidney, liver, and heart [2,3].

SCD has been associated with many cardiovascular complications, including pulmonary hypertension, LV or right ventricular dysfunction, myocardial infarction, decrease in functional capacity, cardiac iron overload, dysrhythmias, and sudden death [4]. The arrhythmias reported with SCD and crisis include QT prolongation, ventricular arrhythmias, first-degree AV blocks, atrial premature contractions, and ventricular premature contractions [4-7].

Many mechanisms can cause chronic dysfunctions in patients with SCD, such as vascular stasis, endothelial activation, nitric oxide depletion, oxidant degeneration, and macrophage activation, causing reperfusion injury [3]. Chronic nitric oxide depletion is associated with vasoconstriction, proliferative vasculopathy, and pulmonary hypertension [3]. LV dilation and diastolic dysfunction are also associated with SCD and are considered to be a common cause of increased morbidity and mortality in patients with SCD [4]. The eccentric dilation of the LV wall and the degree of dilation are related to the severity of anemia [8]. The cause of the myocardial damage is considered due to microvascular disease and iron deposition [4].

A study by Maisel et al. in 30 patients monitored by Holter monitor detected arrhythmias in 80% of patients with sickle cell crisis [5]. A first-degree AV block was noted in 40% of patients with sickle cell crisis. More arrhythmias were found in people with ventricular dysfunction. They also stated that long-term monitoring of patients with sickle cell crisis revealed 80% incidence of arrhythmias compared to 10% by standard EKG.

The mechanism by which these arrhythmias occur is mostly unknown. However, myocardial ischemia associated with sickling during sickle cell crises can be one of the causes of arrhythmias during a sickle cell crisis. However, it has not been substantiated in literature, and further studies need to be conducted to identify the etiology of AV blocks and other arrhythmias.

## Conclusions

Our patient, a young African-American with known SCD and no prior cardiac history, developed an incidental finding of Mobitz type 1 AV block during his admission for an acute sickle cell crisis. It was a transient EKG finding since he was in normal sinus rhythm in EKGs performed in subsequent visits for acute sickle cell crisis. The presence of sinus bradycardia and Mobitz type 1 AV block secondary to high vagal tone in the setting of severe pain in the sickle cell crisis cannot be ruled out. However, there are few case reports and insufficient literature on AV blocks in association with sickle cell crisis, and the presentation of transient second-degree Mobitz type 1 AV block is a rare finding. 
